# Risk Communication and Infodemic Misframing in *Legionella* spp. Environmental Surveillance: An Infodemiology Case Study

**DOI:** 10.3390/microorganisms14030536

**Published:** 2026-02-26

**Authors:** Antonios Papadakis, Eleftherios Koufakis, Nikolaos Raptakis, George Pitsoulis, Apostolos Kamekis, Dimosthenis Chochlakis, Anna Psaroulaki, Areti Lagiou

**Affiliations:** 1 Department of Public and Community Health, University of West Attica, 12243 Athens, Greece; 2 Department of Clinical Microbiology and Microbial Pathogenesis, School of Medicine, University of Crete, Voutes—Staurakia, 71110 Heraklion, Greece; surreydimos@hotmail.com (D.C.); psaroulaki@uoc.gr (A.P.); 3 Public Health Authority of the Region of Crete, 71201 Heraklion, Greece; n.raptakis@crete.gov.gr (N.R.); gpitsoulis@crete.gov.gr (G.P.); kamekis@yahoo.gr (A.K.); 4 Civil Protection of the Region of Crete, 71201 Heraklion, Greece; elkoufakis@crete.gov.gr; 5 Regional Laboratory of Public Health of Crete, School of Medicine, 70013 Heraklion, Greece

**Keywords:** *Legionella*, travel-associated Legionnaires’ disease, infodemiology, malinformation, misinformation, risk communication, Crete, numerical misframing

## Abstract

Travel-associated Legionnaires’ disease (TALD) events can generate public concern when environmental surveillance findings are communicated without an adequate explanation of the results. This study examined how surveillance data on *Legionella* spp. were framed and amplified during a TALD-related investigation in Crete, Greece, from June to July 2025. A mixed infodemiology and environmental surveillance approach was applied, including the analysis of 95 online media items across nine languages, Google Trends search-interest data, and hotel water-system surveillance data from epidemiologically linked facilities. Sampling conducted in a limited number of hotels associated with TALD cases indicated that approximately 50% of the water samples exceeded the laboratory reporting limit of ≥50 CFU/L for *Legionella* spp., a numerically correct but context-specific finding. Numerical misframing occurred in 83.7%, 41.7%, and 18.2% of Greek, German, and English language items, respectively, with significant differences across language markets (χ^2^ (8) = 43.75, *p* < 0.0001; Cramér’s V = 0.679). Public search-interest signals were transient and geographically limited. Environmental surveillance showed no increase in *Legionella pneumophila* risk, with similar proportions of samples ≥50 CFU/L in the pre-/peri-infodemic (January–July 2025) and post-infodemic (August–November 2025) periods (23.11% [95% CI: 18.21–28.87] vs. 24.45% [19.34–30.41]) and similar exceedance of ≥1000 CFU/L (13.45% [9.69–18.36] vs. 14.41% [10.45–19.55]). Overall, the loss of contextual interpretation of surveillance results and conflation of laboratory reporting limits with regulatory thresholds were associated with inconsistent public risk perception, without evidence of increased environmental hazard.

## 1. Introduction

Legionnaires’ disease (LD) is a severe form of pneumonia primarily caused by *Legionella pneumophila*, an aquatic Gram-negative opportunistic bacterium that colonizes engineered water systems and is transmitted through inhalation of contaminated aerosols. Travel-associated Legionnaires’ disease (TALD) presents a distinct public health challenge, as exposure often occurs outside the country of diagnosis, complicating epidemiological attribution and response. Given the ubiquity of *Legionella* spp. in building water systems, particularly in complex tourism-related premises such as hotels, cruise ships, and other large accommodation facilities, where periods of reduced occupancy, limited system operation, and disrupted routine controls during the COVID-19 pandemic favored the development of microbial colonization, the subsequent resumption of normal activity in the post-pandemic period revealed pre-existing system vulnerabilities. In this context, the interpretation of environmental surveillance results is critically dependent on regulatory thresholds, sampling frameworks, and the exposure context, as inadequate communication of these parameters may lead to suboptimal risk management decisions and distorted public perception of risk [1,2,3,4,5,6,7,8,9,10,11,12,13,14,15,16,17].

Beyond microbiological risks and clinical responses, contemporary public health events unfold within an ecosystem of rapid and amplified information circulation. Infodemiology, originally introduced by Eysenbach to describe the study of information distribution and determinants in digital environments, has evolved into a core component of the World Health Organization (WHO) infodemic management framework [18,19,20].

Within this framework, misinformation refers to false or inaccurate information shared without intent to cause harm; disinformation denotes deliberately fabricated or manipulated content; and malinformation describes factually correct information presented without essential contextual qualifiers in ways that mislead public understanding and erode institutional trust. This distinction is particularly relevant for environmental surveillance, where numerically correct findings may become misleading when they are detached from their outbreak-specific sampling frame, a phenomenon commonly described as the loss of the denominator or loss of sampling-frame context [21,22,23,24].

The dynamics discussed are particularly pertinent in regions reliant on tourism, where localized public health incidents can swiftly gain international attention. In these contexts, media amplification may exceed the epidemiological significance of an event, thereby affecting traveler behavior, regulatory measures, and institutional trust. Given its extensive tourism infrastructure and international connectivity, Greece, and specifically Crete, exemplifies a setting in which localized health signals are highly vulnerable to cross-border information amplification [25,26,27,28,29,30].

The miscommunication of risk began after a severe TALD case in Crete in mid-June 2025 (first reported on 14 June 2025), requiring admission to the intensive care unit (ICU) and mechanical ventilation, which attracted extensive media attention. Prior to this revelation, epidemiological investigations had already been conducted in several hotels associated with TALD but had not been made public in order to avoid undue public concern, given that the number of reported cases was comparable to previous years. Following the media leak of the case, public health authorities reported these previously collected findings, including that approximately 50% of the samples exceeded the laboratory reference limit of ≥50 CFU/L. These data were collected as part of case-triggered investigations for *Legionella* spp., and actions were conducted to raise awareness among hotel managers. Although numerically accurate, this context-dependent information was misinterpreted and amplified in media narratives as evidence of widespread contamination, omitting the limited sampling context and confusing laboratory reporting limits with regulatory action limits, thus starting an infodemic [31,32,33,34]. The disclosure of the severe TALD case requiring ICU admission initially generated journalistic and public concern, whereas subsequent numerical amplification emerged only after the public reporting of the “50% positivity” finding.

Crete ranks among Greece’s most prominent tourist destinations, accommodating 1643 hotel units that encompass 196,877 beds and 99,592 rooms, with a significant proportion of these being three-, four-, and five-star establishments. This concentration of large-scale accommodation facilities not only enhances the technical complexity of hotel water systems but also increases the public visibility of health-related signals detected through environmental surveillance, thereby amplifying the potential impact of numerical misframing in public discourse [35,36].

The expansion of this numerically framed narrative extended beyond the media discourse, leading to parliamentary scrutiny and the issuance of a dedicated national circular to clarify the interpretation of surveillance and regulatory practices. Consequently, this episode serves as a well-defined case study to investigate how epidemiologically restricted environmental surveillance findings, referenced in public communication following media reporting of the ICU case, can be transformed into disproportionate public risk narratives through the loss of epidemiological and regulatory contexts. This study aims to (i) characterize the multilingual media amplification and typology of numerical misframing associated with the “50% positivity” narrative, (ii) examine the temporal relationship between media output and public information-seeking behavior, and (iii) contextualize these communication dynamics against longitudinal environmental surveillance data from TALD-linked hotels before and after the infodemic period.

## 2. Materials and Methods

### 2.1. Study Design and Trigger Events

This mixed quantitative–qualitative infodemiology and environmental surveillance study focused exclusively on hotel water systems in Crete, Greece, which were linked to at least one TALD case in 2025 and inspected by the regional public health authorities of Crete following official notification from the European Centre for Disease Prevention and Control (ECDC) and the National Public Health Organization (NPHO).

The trigger event was public reporting of the admission of a traveler with severe TALD to an intensive care unit in Crete (first reported on 14 June 2025). During the same initial reporting window in mid-June 2025, details concerning the patient’s clinical condition, including the need for mechanical ventilation and the severity of illness, as well as, in some instances, visual material, circulated in online news coverage, contributed to heightened public and media attention. Following media disclosure of ICU admission, public reporting referred to environmental inspection findings that had been conducted up to that point as part of routine surveillance, indicating that approximately 50% of collected water samples exceeded the laboratory reporting limit (≥50 CFU/L) for *Legionella* spp. The widespread amplification of the “~50% positivity” message occurred on 18–19 June 2025. These findings were publicly cited in conjunction with the severe clinical outcome, reinforcing the message that increased attention and vigilance were warranted, rather than reflecting results derived from newly initiated inspections. This numerically correct but context-dependent finding was subsequently generalized in public communication and media coverage into broader claims, including assertions that “50% of hotel water in Crete” or “50% of pool water in Crete” was contaminated, and in some reports was incorrectly attributed to domestic split-unit air-conditioning systems, reviving the misnomer “air-conditioner disease”.

### 2.2. Media Dataset

A structured multilingual search strategy was applied using combinations of the terms “*Legionella*”, “Crete”, “hotel(s)”, “pool(s)” and “50%”, and equivalent phrases, using general web search platforms (Google Search and Google News (Google LLC., Mountain View, CA, USA))). For reproducibility, Greek-language searches included the following terms (in Greek script): “Λεγεωνέλλα/Λεγιονέλλα”, “Λεγεωνέλλωση”, “Νόσος των Λεγεωναρίων”, “Κρήτη”, “ξενοδοχείο/ξενοδοχεία”, “πισίνα”, and the phrases “50%” and “50% θετικότητα”. Equivalent terms and translations were used for the other languages represented in the dataset. The primary search window covered 15 June to 15 July 2025, to capture the main amplification burst, and additional eligible items linked to the same event (e.g., later institutional, legal, advisory or explanatory coverage) were included when retrieved during screening and snowballing. The final corpus spanned from 1 April to 1 November 2025.

Items were eligible if they (i) explicitly referred to *Legionella* in hotels or swimming pools in Crete and (ii) either reproduced or interpreted the “50% positivity” figure, or if they provided case-focused, institutional, legal or explanatory coverage of the same event without introducing or repeating quantitative prevalence figures beyond their original, context-restricted reporting. Duplicate URLs, mirrored republications of identical content, and inaccessible paywalled items without full texts were excluded.

**Figure 1:**
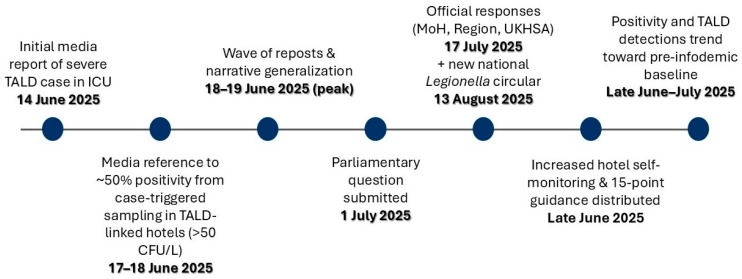
Timeline of key clinical, communication, regulatory, and environmental-investigation events during the 2025 *Legionella* infodemic in Crete, from the initial severe travel-associated Legionnaires’ disease (TALD) case requiring intensive care unit (ICU) admission (first reported on 14 June 2025) to the subsequent numerical amplification of environmental positivity and the return of TALD detections toward pre-infodemic baseline levels. The “50% positivity” figure did not appear in the initial media coverage but emerged during subsequent reporting at the peak of media amplification; the timeline also includes the issuance of a national public health circular on 16 June 2025 aimed at clarifying surveillance interpretation and control measures.

The final, deduplicated corpus comprised 95 unique online items that were locked prior to the analysis. Media reporting, official public health communications, and key regulatory actions were mapped over time and aligned with major milestones (Figure 1). No commercial media monitoring or social listening platforms (e.g., Talkwalker) were used because of resource constraints; only freely available tools (Google Search, Google News, and Google Trends (Google LLC., Mountain View, CA, USA)) and manual keyword-based searches on open online media and social platforms were used. The dataset included publicly accessible posts/pages from open social platforms, treated as ‘online items’ and analyzed using the same codebook.

### 2.3. Infodemic Content Classification and Content Analysis

An operational framework based on the WHO infodemic typology was used to classify the collected media data. Misinformation was defined as inaccurate or false content disseminated without verifiable evidence, where the intent could not be ascertained. Disinformation was defined as apparently false content accompanied by clear evidence of intentional fabrication or manipulation, such as fabricated sources or documents, manipulated graphics, or repeated claims that contradict valid evidence. Malinformation was defined as factually correct or partially correct content that is presented in a misleading manner through omission of essential context, selective reporting, or merging analytical reporting boundaries with normative or action thresholds. As intent cannot be objectively and reliably inferred from published media data, we did not attempt to determine the intent of the article authors. Instead, classification was based on observable content characteristics and verifiability. A decision tree was used for this purpose with the following options: (1) verify the main claim against primary or authoritative sources; (2) if false, it was classified as misinformation, unless there was strong evidence of fabrication or manipulation, in which case classified as disinformation; (3) if realistically correct, assess the completeness of the context, including the sampling frame, denominators, and thresholds; (4) if the key context was missing and the overall message was likely to mislead, classify as malinformation; otherwise, the item was coded as an accurate report. In this setup, misleading owing to missing context corresponds to malinformation.

Qualitative content analysis was performed using a structured codebook adapted from WHO’s infodemic-management typology. Each item was coded separately at the headline and the body-text level into one of four operational categories used in this study: accurate, malinformation (numerically correct claims presented without essential contextual qualifiers, such as the targeted, case-linked sampling frame), misinformation (factually incorrect or misleading statements disseminated without demonstrable intent to deceive), and disinformation (false or misleading content deliberately fabricated, manipulated, or strategically framed with the intent of misleading or cause harm, as evidenced by source patterns, repetition, or coordination) [37,38,39,40].

Additional coding assessed whether items distinguished indicators from regulatory thresholds, acknowledged that the “50% positivity” figure derived from sampling in epidemiologically linked hotels, referred to hotels, pools or both, revived the “air-conditioner disease” misnomer, or cited or contradicted official public health or scientific sources. The codebook was applied by two independent coders following the initial calibration. Intercoder agreement was assessed using Cohen’s kappa coefficient [41].

Given the inability to reliably determine intent from journalistic media content, the disinformation category was considered conceptually but was not applied in the quantitative classification. Importantly, the three 2025 subsets are analytical groupings by time period and investigation context; they do not represent distinct routine surveillance programs, and no repeat follow-up sampling was performed in the same facility.

### 2.4. Published Hotel Surveillance Data

Published environmental surveillance data from hotels in Crete were used to contextualize the reported “50% positivity” figure prior to the infodemic onset. All environmental investigations conducted in 2025 were case-triggered inspections in TALD-associated or epidemiologically linked hotels. The present data support the findings specific to hotels from TALD-associated facilities and are derived from our previous study, which documented *Legionella* spp. colonization patterns in hotels situated in Crete, based on sampling rounds conducted between March 2020 and March 2025.

However, for the purposes of the present analysis, the 2025 environmental dataset was organized into three analytically distinct subsets reflecting the investigation context and timeline: (i) an early-2025 subset from epidemiologically linked hotels (four hotels; 157 water samples), (ii) a pre-/peri-infodemic period (January–July 2025; seven hotels; 238 water samples), and (iii) a post-infodemic period (August–November 2025; 12 hotels; 229 valid water samples).

Accordingly, the published data are referenced here to provide context for positivity patterns in TALD-associated and epidemiologically linked hotels and to clarify that the numerically accurate, yet contextually misinterpreted, “50% positivity” figure is derived from a restricted TALD-linked sampling frame and should not be construed as an estimate of island-wide prevalence.

Each investigation contributed observations from a different hotel; therefore, there was no facility overlap across the three subsets and no repeat sampling at the same facility within the 2025 analytical dataset

### 2.5. Post-Infodemic Hotel Surveillance

Environmental investigations in hotels epidemiologically linked to TALD cases were already ongoing as part of public health response activities and continued throughout 2025, up to November 2025, including during and after the period of intense media attention. The post-infodemic inspections correspond to case-triggered investigations in newly notified TALD-linked hotels (new facilities), rather than follow-up (repeat) sampling in the same hotels. These inspections were not initiated because of the infodemic; rather, they represented the continuation of standard control measures following the TALD case notification.

The resulting post-infodemic analytical subset comprised 229 valid water samples collected from 12 hotels in Crete between August and November 2025. The sampling sites included storage tanks, hot-water boilers, showers, room taps, and pools or spa outlets.

Water samples for microbiological analysis were collected by Regional Public Health Inspectors in sterile 1 L bottles containing sodium thiosulfate to neutralize any residual disinfectant. The samples were transported at a temperature of 5 ± 3 °C and processed within 24 h at the Regional Public Health Laboratory of Crete. On-site measurements of temperature (°C) and free residual chlorine (mg/L) were conducted at the time of sampling using calibrated portable instruments, and the essential descriptors of the sampling points, including location and outlet characteristics, were documented. The microbiological analysis adhered to the ISO 11731:2017 [42] culture method. Water samples were concentrated via membrane filtration, and the concentrates were inoculated onto both selective and non-selective media, followed by incubation at 36 ± 1 °C under humid, CO_2_-enriched conditions. Presumptive *Legionella* colonies were confirmed using standard phenotypic criteria and MALDI-TOF mass spectrometry (MALDI-TOF MS; Bruker Microflex LT, Bruker Daltonics, Leipzig, Germany). Consistent with the laboratory reporting format, the reporting limit was set at 50 CFU/L; thus, positivity was operationally defined as detection at or above 50 CFU/L, with an additional binary variable identifying samples ≥1000 CFU/L, corresponding to the regulatory action threshold [42].

### 2.6. Statistical Analysis and Regulatory/Communication Timelines

Descriptive statistical methods were applied to environmental and infodemiology components. For the environmental datasets, positivity was defined as the proportion of water samples with *Legionella* counts of ≥50 and ≥1000 CFU/L. Wilson 95% confidence intervals (CIs) were calculated for these proportions in the post-infodemic dataset. Given the observational design, comparisons between pre-/peri-infodemic hotel findings (January–July 2025) and post-infodemic sampling were presented using tables and graphical summaries, without multivariable modeling.

For the infodemiology component, media-item frequencies and normalized search-interest indices were summarized descriptively.

Where appropriate, exploratory chi-squared tests were used to compare the prevalence of distorted numerical framing across language groups. Statistical analyses were performed using IBM SPSS Statistics v30.0 (IBM Corp., Armonk, NY, USA) and Epi Info v7.2.7.0 (Centers for Disease Control and Prevention, Atlanta, GA, USA).

## 3. Results

### 3.1. Media Coverage of the “50% Positivity” Narrative

A structured multilingual web search (Google Search/Google News) identified 95 distinct online items in nine languages that reproduced or contextualized the “50% positivity” narrative following the hospitalization of a British tourist in the ICU in Crete, Greece, between 15 June and 1 November 2025. The body of text was primarily composed of Greek-language sources, which also had the highest incidence of misinformation, while English and German-language items were largely accurate with minimal numerical distortion. Overall, 51.6% of the items were classified as accurate, 43.2% as malinformation, and 5.3% as misinformation. Other languages showed limited adoption of the infodemic and were mostly accurate. Some publications, mainly from the United Kingdom, also included patients’ personal or medical details and, in some cases, photographs of the patient. The details of multilingual enhancement and classification across languages, including distorted framing and peak publication dates, are presented in Table 1.

In addition, a descriptive manual audit of mainstream online news coverage during June 2025 identified 25 distinct items (17 Greek, 8 English), with a pronounced clustering during 18–19 June 2025 (>12 items, approximately 50% of the audited set). Even in items that mentioned clinical updates regarding the patient, the dominant audience-facing framing foregrounded the environmental “~50% positivity” narrative rather than patient-centered clinical severity.

### 3.2. Temporal Amplification Dynamics

Media activity showed a highly concentrated early burst, with a clear language-specific timing pattern. In the Greek language corpus (EL), publication volume peaked on 19 June 2025 (*n* = 28 items/day), preceded by less activity on 17–18 June (*n* = 4 items/day), followed by residual output on 20 June 2025 (*n* = 3). German language coverage (DE) peaked on 22–23 June 2025 (*n* = 2 items/day). English language coverage (UK) exhibited a short-lived burst peaking on 20 June 2025 (*n* = 5 items/day), with a secondary rise on 23 June 2025 (*n* = 4 items/day), patient-centric spillover rather than sustained numerical pickup.

When aligned with Google Trends peak search interest (used here as a proxy of audience attention/“readership”), the search peak in Greece occurred four days after the Greek media peak (23 June 2025, vs. 19 June 2025), suggesting delayed conversion of media exposure into active information seeking. In Germany, the search peak (22 June 2025) coincided with the onset of the German media peak window (22–23 June 2025), indicating a near-synchronous amplification. In the UK, search interest peaked substantially later (5 July 2025), that is, 15 days after the English language media peak (20 June 2025), which is consistent with the attention driven by subsequent case-related developments rather than the numerical claim itself (Table 2).

### 3.3. Source Categorization Across Infodemic Typology Categories

Across the corpus, malinformation was characterized by the reproduction of the “50%” figure without explicit reference to the targeted sampling conducted in epidemiologically linked hotels. At the title level, titles were 61.1% accurate (58/95), 34.7% malinformation (33/95), and 4.2% misinformation (4/95), whereas content level classification showed 53.7% accuracy (51/95), 43.2% malinformation (41/95), and 3.2% misinformation (3/95).

Headline and body-text classifications were concordant for of 82/95 (86.3%) items. Discordance occurred in 13/95 (13.7%), most commonly reflecting accurate headlines paired with malinformative body text (eight items), indicating that contextual qualifiers in titles did not consistently carry into the narrative framing of the entire article (Table 3).

### 3.4. Cross-Language Distortion Pattern

Cross-language dissemination showed a clear gradient in the prevalence of distorted numerical framing across language groups. Distorted numerical framing (malinformation plus misinformation; “worst case” item-level classification) was the highest in the original language market (Greek: 36/43, 83.7%), decreased in German (5/12, 41.7%), and was comparatively limited in English language coverage (UK: 4/22, 18.2%) and Hebrew-language coverage (1/4, 25.0%). In the remaining languages represented in the corpus (Italian, French, Danish, Norwegian, and Swedish), no distorted numerical framing was observed (0%), with the items being predominantly explanatory or case-focused. English dissemination remained largely patient-centric, with 81.8% of the UK items classified as accurate, omitting the “50%” claim.

**Figure 2:**
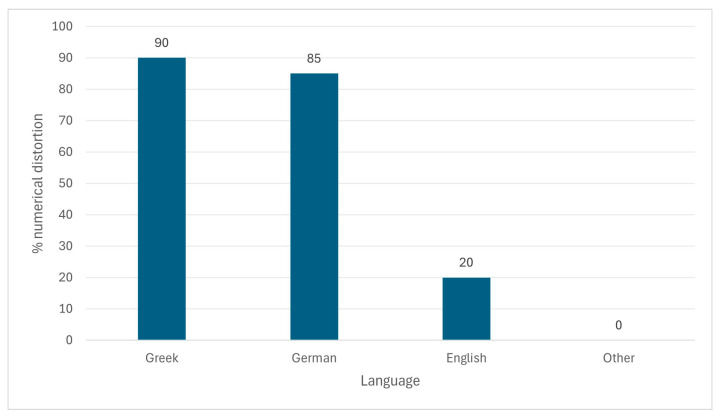
Proportion of media items exhibiting numerical distortion, stratified by language. The bar chart illustrates categorical differences between language groups and does not imply temporal ordering or continuity.

As illustrated in Figure 2 and supported by the Google Trends timing analysis in Table 2, audience attention (search interest) followed media activity with market-specific lags (Greece: +4 days; Germany: 0 days; UK: +15 days) [43]. In addition, Google Trends indicated no measurable worldwide search interest for the numerical narrative query analyzed (Table 2/Trends analysis), despite the volume of online items.

A chi-square test confirmed that the prevalence of distorted numerical framing differed significantly across languages (χ^2^ (8) = 43.75, *p* < 0.0001; Cramér’s V = 0.68), indicating a statistically significant association between language group and the prevalence of distorted numerical framing.

### 3.5. August–November 2025 Hotel Surveillance Findings

Environmental investigations of hotels epidemiologically linked to TALD cases continued during and after a period of intense media attention. In the post-infodemic analytical subset, 229 valid water samples were collected from 12 hotels in Crete between August and November 2025, as part of the ongoing public health control activities in epidemiologically linked hotels.

In the August–November 2025 subset, 24.45% of samples were reported at or above the laboratory reporting limit, and 14.41% exceeded the legislated threshold. These values were comparable to those observed in the January–July 2025 period and substantially lower than those of the early 2025 epidemiologically linked subset, in which approximately 59% of samples exceeded the laboratory reporting limit. A comparative overview of the three 2025 analytical subsets is presented in Table 4.

**Figure 3:**
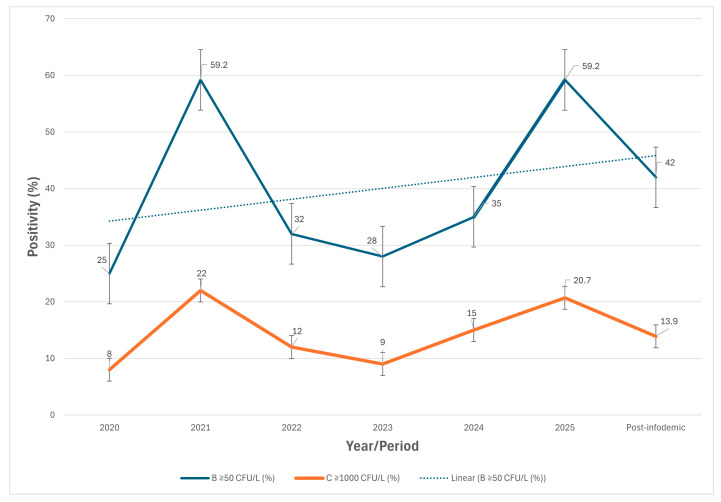
Annual *Legionella* positivity (≥50 CFU/L) in hotel water samples from Crete, 2020–2025, with an additional post-infodemic sampling point.

As illustrated in Figure 3, *L. pneumophila* positivity showed pronounced peaks during periods of intensified investigation in epidemiologically linked hotels, followed by a return to the baseline levels in the annual series. The inclusion of 2021 data serves a comparative purpose, illustrating that similarly high environmental positivity levels have been observed previously during TALD-linked surveillance, without subsequent public disclosure or media amplification.

### 3.6. Microbiological and Physicochemical Risk Characteristics

Comparison of microbiological and physicochemical indicators before and after infodemic showed largely stable colonization patterns. The detection of *L. pneumophila* serogroup 1 remained low, whereas serogroups 2–15 predominated in both periods, with overlapping confidence intervals.

Thermal control showed a partial improvement, with reductions in the proportion of hot water outlets below 55 °C and cold water outlets above 25 °C. In contrast, the proportion of outlets with free residual chlorine < 0.2 mg/L increased, likely reflecting frequent flushing and hydraulic disturbance during remedial actions.

A detailed comparison of microbiological and physicochemical risk indicators before and after the infodemic period is presented in Table 5.

### 3.7. Institutional Response

Following the media coverage of the “50% positivity” figure, national and regional public health authorities issued official communications and notices. These communications specified that the reported figure was based on environmental investigations conducted in epidemiologically linked hotels and did not represent population-level surveillance.

On 16 June 2025, a national circular entitled “Reminder of the circular on public health protection measures against Legionnaires’ disease” was issued, addressed to inspection and public-health control services [44].

The circular indicated that precautionary measures and control actions may be implemented based on documented risk assessment, even in the absence of positive microbiological findings. International public health advisories, including those issued by UK authorities, focused on case detection and accommodation exposure history, without reference to generalized positivity rates.

## 4. Discussion

The core mechanism involves a transition from case-triggered findings to population-level risk narratives. In the present case study, this transition occurred when a numerically accurate laboratory statement (“~50% of samples ≥50 CFU/L”) derived from a restricted, TALD-linked, case-triggered sampling frame was separated from its denominator and sampling principle. It was subsequently disseminated as if it represented island-wide environmental conditions. This “loss of sampling-frame context” was worsened by confusing thresholds; the laboratory reporting limit (used to report detectable growth) was frequently mistaken as a regulatory exceedance threshold, thereby promoting a population-level hazard narrative. Importantly, public reporting of the patient’s severe clinical course was factual and not itself misinformation; the infodemic distortion arose from the misframing and overgeneralization of context-dependent environmental surveillance findings (the “~50% positivity” narrative). Consistent with our results, even in online news items that referenced the patient’s clinical condition, the dominant audience-facing emphasis foregrounded the environmental “~50% positivity” narrative rather than patient-centered clinical reporting.

Three distinct levels of risk were conceptually included in this study: (i) laboratory risk (whether *Legionella* is detected at or above the reference limit), (ii) regulatory risk (whether concentrations exceed action limits requiring specific control measures), and (iii) perceived/social risk (how the public interprets the signal and modifies behavior). During the amplification episode, communication often bypassed the regulatory level (thresholds, actions, and surveillance mandates triggered by the case), moving directly from laboratory risk to perceived or social risk, thus facilitating misinterpretation.

Effective management of TALD events depends on accurate interpretation of environmental surveillance findings and proportionate, context-aware communication. The Cretan infodemic illustrates an epidemiologically restricted observation—approximately 50% of samples exceeding the laboratory reporting limit in a limited number of TALD-linked hotels—was rapidly transformed into generalized narratives suggesting widespread contamination across Crete. This represents a phenomenon, whereby case-linked findings are misinterpreted as population-level evidence when detached from the sampling frame [45,46,47,48,49,50,51,52,53].

Within the WHO infodemic-management framework, this pattern is best characterized as malinformation: correct data presented without essential contextual qualifiers. In the present case, numerical exceedance at the laboratory reporting level was repeatedly conflated with the exceedance of legislated thresholds, erasing the regulatory distinction that underpins risk-based decision making for *Legionella* control. This example demonstrates how laboratory reporting (detection) can be misinterpreted as regulatory exceedance, subsequently leading to the perception of social risk without the necessary regulatory interpretation. Rather than reiterating the definitions provided in the Methods section, these findings highlight the operational consequences of such conflation, particularly within Greek language media and, to a lesser extent, in secondary German-language amplification [54,55,56,57,58,59,60,61,62,63,64,65,66].

Tourism-dependent regions such as Crete are especially vulnerable to this form of numerical misframing. Localized public health signals can rapidly acquire international visibility, with media amplification extending well beyond the epidemiological significance of the initiating event. In the present case, English-language coverage, largely oriented toward the clinical course of the affected traveler, showed substantially lower numerical distortion, underscoring how market proximity, language, and audience framing shape infodemic dynamics [67,68].

An additional infodemic dimension observed during this episode concerned breaches of medical confidentiality in the public discourse. The current legislative framework and good risk communication practices require that when investigating TALD cases, established confidentiality protocols are strictly followed during the notification of the TALD, limiting information about the cases to non-identifiable epidemiological indicators, such as country of origin, age group, gender, length of stay, and clinical condition, and when receiving the findings of the investigation and risk assessment control. On the other hand, it must be emphasized that the absence of information such as the room of stay limits the more correct sampling of the associated hotel. Despite the requirements of this confidentiality, in present study, many online media outlets disseminated unauthorized medical data about the hospitalized tourist. Coverage in Greek often included detailed information about the patient’s clinical condition, while English coverage often included additional visual material, such as photographs of the patient. This equally significant information leak demonstrates how infodemics can extend beyond numerical misrepresentation of environmental surveillance data to include breaches of patient privacy, especially at travel-related events of high interest to prospective visitors to the area in question. Such practices risk increasing public anxiety, causing cancelations of trips or changes in accommodation, and generally undermining trust in the management of public health protection by the relevant authorities by shifting public attention from population-level risk management to personalized and sensational narratives.

Longitudinal environmental surveillance data from TALD-associated hotels provides an essential context for interpreting narrative distortion. As previously documented by Papadakis et al. (2025), *Legionella* positivity in hotel water systems exhibits the expected temporal variability, with pronounced peaks during case-triggered investigations and reopening-related stagnation phases. Outside these periods, the surveillance indicators remained substantially lower and stable. Within this established framework, the widely publicized “~50% positivity” figure corresponds to a limited case-related subset rather than baseline or island-wide environmental conditions [9,33,69].

The central question addressed by this study was whether the infodemic itself translated into measurable improvements in environmental *Legionella* outcomes. A comparison of pre- and post-infodemic hotel surveillance data did not support a reduction in microbiological positivity. Exceedance proportions before and after the infodemic period showed overlapping confidence intervals, suggesting broadly similar proportions across periods (descriptive comparison). These findings suggest that heightened public and media attention should not be interpreted as proxies for improved environmental outcomes.

One possible explanation is that increased attention may lead to short-term, non-sustained corrective actions (e.g., one-time thermal shock, intensive flushing, ad hoc disinfection) that do not result in long-term control of colonization. Achieving sustainable improvement typically dictates system-level implementation and verification of a comprehensive Water Safety Plan, comprising risk assessment, control measures, monitoring, documentation, and continuous management, rather than relying on episodic, “one-off” interventions.

However, instances where regulatory limits were exceeded indicate compliance or non-compliance with an otherwise flexible regulatory framework and are not assessed as a deterioration of environmental conditions. The expected compliances in the parametric temperature values of the distribution water (hot and cold) are consistent with the increased management and compliance efforts with the regulations. The reduced presence of free residual chlorine is probably attributed to frequent flushing, temporary plumbing maintenance work, and customer pressure for more environmentally friendly disinfectants and to the absence of residual chlorine, which is easily recognized by its odor, especially in foreseen and required shocks. Combined, these trends indicate preventive risk management rather than a systematic non-compliance of hotel water systems, always underlining that preventive measures do not directly lead to microbiological improvements but, on the contrary, full compliance eliminates the risk of possible re-colonization [56,57,58,59,70].

The infodemic also revived persistent misconceptions regarding the *Legionella* transmission pathways. Several media reports incorrectly attributed the risk to domestic split-unit air-conditioning systems, reviving a long-standing misinterpretation of the historical term ‘air-conditioner disease’. Such misattributions divert attention from established high-risk water-based systems and may undermine effective prevention strategies by focusing on public concerns regarding irrelevant exposure routes [71,72,73,74,75,76,77,78,79].

Notably, references to transmission routes such as split-unit air-conditioning systems appeared primarily in televised discussions rather than in systematically retrievable online media items and therefore could not be captured or quantitatively analyzed within the media corpus. Nonetheless, these statements reflect recurrent gaps in public understanding of *Legionella* transmission that have also been documented in previous episodes following the public disclosure of severe hospitalization or death.

From a governance perspective, the episode triggered substantial institutional responses, including parliamentary scrutiny and coordinated clarification by the national and regional authorities. Official communications and a subsequent national circular explicitly reintroduced the sampling frame and regulatory context, reaffirming that precautionary measures may be implemented based on documented risk assessment, even in the absence of positive microbiological findings. These actions illustrate the contrast between structured public health communication and generalized numerical claims disseminated through media narratives.

A critical reflection pertains to the initial public dissemination of the “~50%” figure. Even when the objective is to encourage awareness, presenting a numerator without simultaneously and explicitly specifying the denominator (total number of samples), the limited TALD-linked sampling mandate, and the differentiation between reporting limits and regulatory thresholds creates conditions contributing to misinterpretation. Providing the denominator and sampling principle at the initial mention would likely have constrained the interpretive scope for generalized “island-wide contamination” narratives and may have mitigated subsequent distortion.

Overall, this case highlights the critical relationship between environmental surveillance and public communication. Proactive, communication-driven governance actions may be misinterpreted as widespread risk unless technical findings, limits, and justifications for decisions are communicated transparently and consistently. Without such clarity, technically valid surveillance outputs risk evolving into infodemics that erode institutional trust without reflecting true changes in environmental hazard.

In highly touristed settings, reputational risk can function alongside health risk: even a localized, case-triggered surveillance signal may affect traveler perceptions and stakeholder behavior when presented as a population-level threat. Although we did not have access to detailed tourism-economy indicators (e.g., bookings, cancelations, revenue) to quantify this impact in the current study, the observed multilingual media coverage and temporary search-interest signals support a plausible pathway through which numerical misrepresentation can exert economic and reputational pressure that is disproportionate to documented environmental findings.

### 4.1. Limitations

This study has several limitations. Although the body of media is multilingual, it is limited to freely accessible online sources, potentially excluding private, commercial, or subscription-based content. Sampling was limited to hotels that were epidemiologically linked to TALD cases, which may limit the generalizability of the environmental findings to the wider island context. Furthermore, media item classification was based on a predefined typology, which may introduce subjectivity despite structured coding.

### 4.2. Recommendations/Future Studies

Future research should examine both automated and large-scale informatics methods to numerically monitor incorrect claims about environmental surveys and simultaneously monitor privacy violations in real time. A key priority for central and regional public health authorities and epidemiologists is to prioritize transparent and informed risk communication, distinguishing laboratory reporting limits from regulatory action thresholds. National and regional public health authorities should anticipate the potential for infodemics, especially at high-profile travel-related events, and incorporate ethical safeguards to protect individual privacy while maintaining effective population-level risk communication with scientific updates increasingly understandable to non-specialist citizens on social media. Infodemiology metrics should also be integrated with tourism indicators to quantify this mechanism. Finally, future research should investigate not only news portals but also the role of social media ranking and recommendation algorithms in influencing the visibility and cross-language dissemination of the “50%” figure. Additionally, it should assess whether early “denominator-first” public communication can significantly mitigate the amplification dynamics.

## 5. Conclusions

The *Legionella* “infodemic” in Crete during June and July 2025 was local and short-lived, but it highlighted that environmental surveillance findings can be amplified into population-level risk narratives when they are integrated into preventive public communication without adequate clarification of the sampling and regulatory framework. In tourist areas, such amplification extends beyond public health impacts and can cause reputational pressures—not only for the area itself but also in behavioral responses disproportionate to documented environmental risk. The systematic use of social listening tools, combined with complementary platforms such as Google Trends, is critical for public health authorities to assess the societal impact of emerging narratives in real time and limit escalation through timely and proportionate communication.

In accordance with best practice in risk communication, when reporting high positivity rates from case-triggered, TALD-associated environmental investigations, public health authorities should present both the numerator and denominator, such as the number of positive samples, the total number of samples, and the number of facilities or establishments. They should also explicitly state why sampling was conducted and distinguish laboratory reporting or reporting limits from regulatory action limits.

The results of the study reconfirmed that effective prevention of *Legionella* infection cannot be based on microbiological results alone. Environmental data from microbiological analyses below the reference limits set by regulations or the laboratory limit do not indicate the absence of risk due to the intermittent nature of colonization and the limitations of spot sampling. Written and documented risk assessments, together with Water Safety Plans, remain critical tools for effective risk management.

Finally, the findings of this study and of similar published TALD studies indicate that cases are predominantly attributed to *L. pneumophila*. Therefore, maintaining a single regulatory threshold generally stated for *Legionella* spp. should be reconsidered, as it may lead to unnecessary costs, repeated sampling, increased administrative/operational burden, and risk communication practices that emphasize numerical exceedance rather than the epidemiological significance of the findings.

## Figures and Tables

**Table 1:** Multilingual amplification of the narrative of “50% positivity” (*n* = 95).

Language	Sites (*n*)	Distorted Framing *n* (%)	Malinformation *n* (%)	Misinformation *n* (%)	Accurate *n* (%)	Peak Publication Date(s) (2025) *
Greek	43	36 (83.7%)	32 (74.4%)	4 (9.3%)	7 (16.3%)	19 June 2025
English (UK unified)	22	4 (18.2%)	3 (13.6%)	1 (4.5%)	18 (81.8%)	20 June 2025
German	12	5 (41.7%)	5 (41.7%)	0 (0.0%)	7 (58.3%)	22 June 2025, 23 June 2025
Italian	5	0 (0.0%)	0 (0.0%)	0 (0.0%)	5 (100.0%)	1 July 2025
Hebrew	4	1 (25.0%)	1 (25.0%)	0 (0.0%)	3 (75.0%)	21 June 2025
Danish	3	0 (0.0%)	0 (0.0%)	0 (0.0%)	3 (100.0%)	23 June 2025, 24 June 2025, 25 June 2025
French	3	0 (0.0%)	0 (0.0%)	0 (0.0%)	3 (100.0%)	1 April 20251 May 2025
Norwegian	2	0 (0.0%)	0 (0.0%)	0 (0.0%)	2 (100.0%)	1 June 2025, 1 November 2025
Swedish	1	0 (0.0%)	0 (0.0%)	0 (0.0%)	1 (100.0%)	n/a
Total	95	46 (48.4%)	41 (43.2%)	5 (5.3%)	49 (51.6%)	—

* Peak publication date(s) = date(s) with the highest within-language item count in 2025 (ties shown as ranges).

**Table 2:** Temporal synchronization between media peaks and search interest.

Market/Language Group	Media Peak Date (from Corpus)	Media Peak Intensity (Max Items/Day)	Google Trends Query (as Analyzed)	Trends Peak Date	Lag (Trends Peak–Media Peak), Days
Greece/Greek (EL)	19 June 2025	28	“50% positivity Crete” (Greek-language query)	23 June 2025	+4
Germany/German (DE)	22 June 2025 to 23 June 2025	2	“*Legionella* Kreta”	22 June 2025	0
UK/English (UK)	20 June 2025	5	“*Legionella* Crete”	5 July 2025	+15

**Table 3:** Title vs. content distortion analysis (full corpus, *n* = 95).

Metric	Accurate	Malinformation	Misinformation
Title Category (Recoded)	58 (61.1%)	33 (34.7%)	4 (4.2%)
Content Category (Recoded)	51 (53.7%)	41 (43.2%)	3 (3.2%)
Concordance (Title = Content)	82/95 (86.3%)		
Discordance (Title ≠ Content)	13/95 (13.7%)		

**Table 4:** *Legionella* colonization in hotel water systems before and after the infodemic (Crete, 2025).

Dataset/Period	Hotels (*n*)	Samples (*n*)	≥50 CFU/L	95% CI	≥1000 CFU/L	95% CI	Key Notes
Early 2025 subset from epidemiologically linked hotels	4	157	59.23%	51.42–66.61%	7.64%	4.43–12.88%	Sampling in epidemiologically linked hotels; not population-level
Pre-/peri-infodemic January–July 2025	7	238	23.11%	18.21–28.87%	13.45%	9.69–18.36%	Passive, case-linked investigations
Post-infodemic August–November 2025	12	229	24.45%	19.34–30.41%	14.41%	10.45–19.55%	Continued investigations; remedial actions

Overlapping 95% confidence intervals between the January–July 2025 and August–November 2025 subsets are shown for descriptive context and should not be interpreted as formal hypothesis testing; overall positivity at the ≥50 CFU/L reporting limit appeared broadly similar across periods.

**Table 5:** Microbiological and physicochemical indicators before vs. after the infodemic.

Indicator	Pre-Infodemic (*n* = 238)	95% CI	Post-Infodemic (*n* = 229)	95% CI	Interpretation
*L. pneumophila* SG1	5.88%	3.25–9.67%	3.93%	1.81–7.33%	Stable, low
*L. pneumophila* SG2–15	17.23%	12.92–22.57%	19.65%	14.69–25.59%	Stable
Hot water < 55 °C	79.80%	73.96–84.53%	65.30%	57.57–72.40%	Lower (descriptive)
Cold water > 25 °C	54.31%	44.81–63.59%	48.11%	38.30–58.03%	Lower (descriptive)
Free chlorine < 0.2 mg/L	15.74%	9.45–24.00%	31.46%	22.03–42.17%	Higher non-compliance (descriptive)

## Data Availability

The data presented in this study are available on request from the corresponding author. The data are not publicly available due to privacy and confidentiality restrictions.

## Abbreviations

CI	Confidence interval
CFU	Colony-forming units
ECDC	European Centre for Disease Prevention and Control
ICU	Intensive Care Unit
ISO	International Organization for Standardization
LD	Legionnaires’ disease
NPHO	National Public Health Organization (Greece)
SG	Serogroup
TALD	Travel-associated Legionnaires’ disease
UKHSA	UK Health Security Agency
WHO	World Health Organization
